# Invasive Fungal Disease, Isavuconazole Treatment Failure, and Death in Acute Myeloid Leukemia Patients

**DOI:** 10.3201/eid2509.190598

**Published:** 2019-09

**Authors:** Anne-Pauline Bellanger, Ana Berceanu, Emeline Scherer, Yohan Desbrosses, Etienne Daguindau, Steffi Rocchi, Laurence Millon

**Affiliations:** University Hospital, Besançon, France (A.-P. Bellanger, E. Scherer, S. Rocchi, L. Millon);; Franche-Comté University, Besançon (A.-P. Bellanger, E. Scherer, L. Millon);; Besançon University Hospital, Besançon (A. Berceanu, Y. Desbrosses, E. Daguindau)

**Keywords:** aspergillosis, fungal co-infection, invasive fungal disease, isavuconazole, treatment failure, mucormycosis, quantitative PCR, qPCR, computed tomography, CT, graft-versus-host disease, *Aspergillus fumigatus*, *Rhizomucor*, France, antimicrobial resistance, fungi, acute myeloid leukemia, hematopoietic stem-cell transplantation, galactomannan antigen, immunocompromised patients, death

## Abstract

We present 2 fatal cases of invasive fungal disease with isavuconazole treatment failure in immunocompromised patients: one with a TR34-L98H azole–resistant *Aspergillus fumigatus* isolate and the other a *Rhizomucor*–*A. fumigatus* co-infection. Such patients probably require surveillance by galactomannan antigen detection and quantitative PCRs for *A. fumigatus* and Mucorales fungi.

Isavuconazole, an antifungal azole used to treat invasive fungal diseases (IFDs), is approved as a first-line treatment for invasive aspergillosis and can be used as an alternative treatment for mucormycosis (*1*). We present 2 cases of IFD and isavuconazole treatment failure in acute myeloid leukemia (AML) patients with prolonged neutropenia after hematopoietic stem-cell transplantation (SCT).

Patient 1, a 52-year-old truck driver with AML (diagnosed in 2017), received a haplo-identical SCT (day 0) 4 months after the diagnosis. The patient had incomplete hematologic reconstitution and experienced graft-versus-host disease of the digestive tract (day 0), which we treated with corticosteroids and ruxolitinib. We gave the patient oral posaconazole (300 mg/day, starting day 1) for IFD prophylaxis, as recommended by the 4th European Conference on Infections in Leukaemia (*2*). On about day 65, we diagnosed probable invasive aspergillosis according to the criteria of the European Organisation for Research and Treatment of Cancer Mycoses Study Group (*3*,*4*); the patient had fever, neutropenia, and a discrete pulmonary lesion on chest computed tomography (CT), and serum samples were repeatedly positive by an in-house *A. fumigatus* quantitative PCR (qPCR) but negative for galactomannan antigen (Figure) (*1*,*4*). On the same day, we switched treatment to oral isavuconazole (200 mg/day). On day 126, chest CT showed the persistence of pulmonary lesions, and we switched patient treatment to liposomal amphotericin B (5 mg/day by injection). Thereafter, serum samples became repeatedly positive for galactomannan antigen and *A. fumigatus* DNA. At day 158, we found TR34-L98H azole–resistant *A. fumigatus* fungus in his bronchial aspirate. The French National Reference Center for Invasive Mycoses and Antifungals (Paris, France) performed MIC testing using European Committee on Antibiotic Susceptibility Testing methods (https://www.pasteur.fr/fr/sante-publique/CNR/les-cnr/mycoses-invasives-antifongiques). The following MICs were obtained: amphotericin B 0.25 mg/L (susceptibility unknown, no breakpoint available), itraconazole >8 mg/L (resistant), isavuconazole 4 mg/L (resistant), and voriconazole 2 mg/L (susceptible). The patient died 182 days after the SCT.

**Figure:**
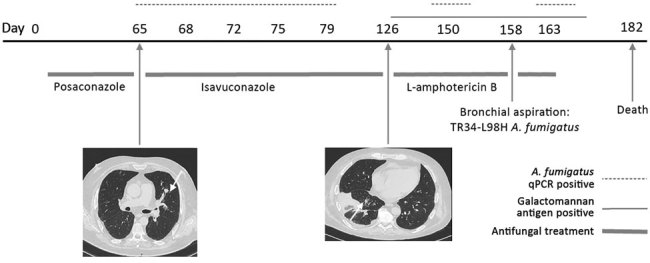
Evolution of fungal biomarkers, computed tomography chest scans, and antifungal treatments for immunocompromised patient 1 with invasive *Aspergillus* *fumigatus* infection, France, 2018. Arrows indicate lesions. qPCR, quantitative PCR.

Patient 2, a 61-year-old businessman with AML (diagnosed in 2013), received his first allogenic hematopoietic SCT 4 months after the diagnosis. He experienced a relapse 3 years after the first transplantation, and a second allogenic hematopoietic SCT was performed (day 0), which was followed by severe sepsis with *Escherichia coli*. An excavated nodule was visible on chest CT (day 4), and the *A. fumigatus* biomarker was repeatedly positive, suggesting probable invasive aspergillosis according to European Organisation for Research and Treatment of Cancer criteria (*3*). On day 5, treatment with isavuconazole (200 mg/day orally) was initiated. Graft-versus-host disease of the digestive tract also developed (day 12) in this patient, which we treated with ruxolitinib, tacrolimus, and prednisolone. On day 92, the patient had asthenia, fever, and thoracic pain, and chest CT showed multiple micronodules. On days 95–116, systematic fungal surveillance testing of serum samples showed 1 test positive for galactomannan antigen, 4 positive for *A. fumigatus* DNA, and 3 positive for *Rhizomucor* DNA. Two cultured pulmonary samples collected on days 114 and 116 were positive for *A. fumigatus*. We performed ETESTs (bioMérieux, https://www.biomerieux-diagnostics.com), which indicated the following MICs: amphotericin B 0.023 mg/L (susceptibility unknown), isavuconazole 0.25 mg/L (susceptible), and voriconazole 0.38 mg/L (susceptible). We switched patient treatment to liposomal amphotericin B (5 mg/kg by injection) on day 117, but the patient died on day 129.

These 2 cases had in common AML treated by SCT, followed by severe digestive graft-versus-host disease, IFD resistant to isavuconazole diagnosed >100 days after SCT, use of combined fungal biomarkers to detect IFD, and death despite rapid prescription of amphotericin B. Severe digestive graft-versus-host disease might have affected the levels of isavuconazole absorbed by the patient because the drug was administered orally in both cases. Intravenous isavuconazole is not recommended for treating IFD and was not available at the treatment facility (University Hospital, Besançon, France). However, these cases suggest that isavuconazole levels should be checked in patients with severe digestive graft-versus-host disease. For patient 1, a change in class of antifungal drugs could have been made as early as day 65, and earlier treatment with amphotericin B could have had a positive effect on his prognosis. The long duration between the initial positive qPCR and galactomannan antigen test result suggests patient 1 might have been infected with multiple *A. fumigatus* isolates, with 1 being resistant. For patient 2, the systematic use of Mucorales qPCR enabled early detection of a mixed *Aspergillus*-Mucorales fungal infection (*5*). These types of mixed mold infection were reported to have a prevalence of 25% in studies including Mucorales qPCR (*6*). The diagnosis of *Rhizomucor* infection was based on 3 successive samples being positive by *Rhizomucor* qPCR. A Mucorales qPCR of the patient’s bronchoalveolar lavage fluid (validated assay with satisfying sensitivity) was surprisingly negative (*6*).

In summary, these cases demonstrate that systematic surveillance is needed for severely immunocompromised patients treated for IFD. Galactomannan antigen detection and qPCRs targeting *Aspergillus fumigatus* and Mucorales fungi might be the optimal surveillance strategy.
